# Exposure to a nicotinoid pesticide reduces defensive behaviors in a non-target organism, the rusty crayfish *Orconectes rusticus*

**DOI:** 10.1007/s10646-018-1950-4

**Published:** 2018-05-25

**Authors:** Lauren Sohn, Renae J. Brodie, Genevieve Couldwell, Eleanor Demmons, Joachim Sturve

**Affiliations:** 1 Department of Biological Sciences, Mount Holyoke College, South Hadley, Massachusetts 01075 USA; 2 Department of Biological and Environmental Sciences, University of Gothenburg, Göteborg, 40530 Sweden

**Keywords:** Imidacloprid, Sublethal effects, Chronic toxicity, Crayfish, Behavior

## Abstract

Imidacloprid is the most widely used of the nicotinoid insecticides, the fastest growing class of pesticides on the global market. Although less toxic to mammals and birds compared to organophosphates, nicotinoids have the potential to impact non-target invertebrates, especially through sublehal effects on behavior, physiology, reproduction, and development. We investigated the impact of sublethal doses of imidacloprid on the defensive responses of rusty crayfish *Orconectes rusticus* exposed to 0, 1, 10, and 100 µg•L^−1^ of imidacloprid for 10 days (*n* = 7 crayfish per treatment). Defensive behaviors were examined with the rod test, in which a glass rod was jabbed into the crayfish’s container at a 90 degree angle from the bottom and about 0.5 cm directly in front of the crayfish. Crayfish responded to the rod aggressively with claw raising and pinching, neutrally (no response), or by backing or tail-flipping away. The frequency of neutral responses more than doubled after four days in the high (100 µg•L^−1^) group and after eight days in the low (1 µg•L^−1^) exposure group. Furthermore, most crayfish in the 100 µg•L^−1^ treatment were not able to right themselves within 30 s when placed on their backs. Several studies have reported concentrations of imidacloprid contamination in freshwater ecosystems that exceed this study’s lowest exposure scenario, 1 µg•L^−1^. We therefore conclude that imidacloprid contamination reduces the defensive behaviors of crayfish, impairing their ability to survive in habitats where they play important ecological roles.

## Introduction

In 2000, as the organophosphorus pesticides diazinon and chlorpyrifos were phased out of urban use in the United States, the US Environmental Protection Agency suggested replacing them with nicotinoids. Nicotinoids are now the fastest growing class of insecticides world-wide, with the largest market share going to imidacloprid (Nauen et al. 2008; Jeschke et al. 2011), a pesticide primarily used as a seed dressing to manage arthropod crop pests and in topical flea and tick control treatments for pets (Casida 2018). Engineered to function as nicotinic acetylcholine receptor (nAChR) agonists that disrupt synaptic transmissions (Buckingham et al. 1997; Tomizawa and Casida 2005; Charpentier et al. 2014), nicotinoids show a higher affinity for arthropod compared to vertebrate nAChRs, making them safer for humans (Feng et al. 2004; Matsuda et al. 2001; Moffat 1993).

While imidacloprid is attractive because of its relatively low toxicity to humans, it poses a threat to non-target organisms, including those occupying aquatic habitats for part or all of their lifecycles. Imidacloprid has a half-life of one to six months in soil, depending on temperature and pH conditions (Wood and Goulson 2017; Sarkar et al. 2001; Scholz and Spiteller 1992), and is highly soluble in water (0.61 g•L^−1^, 20 °C; Tomlin 1997). Runoff from crops during rainfall in the planting season is the greatest source of contamination for aquatic ecosystems (Wood and Goulson 2017), but groundwater seepage, dust, and uprooted plants grown from prophylactically treated seeds also contribute (Bonmatin et al. 2015; Douglas and Tooker 2015). In Canada, concentrations of 11.9 µg•L^−1^ were measured in runoff from potato fields during rain events (CCME 2007), while concentrations of 1–14 µg•L^−1^ have been measured in surface and groundwater in the USA (Jemec et al. 2007; references therein), and levels up to 320 µg•L^−1^ have been reported from monitoring sites in agricultural areas in the Netherlands according to the Dutch Pesticide Atlas (as cited in van Dijk et al. 2013). World-wide, a mean value of 0.13 µg•L^−1^ was reported from surface water monitoring sites from nine countries (Morrissey et al. 2015).

Imidacloprid concentrations in the 1–15 µg•L^−1^ range found in the more contaminated freshwater sites in the US and Canada are not lethal to most aquatic organisms (but see Raby et al. 2018 and van den Brink et al. 2016 for data on sensitive larval insects)—e.g., the 48-h LC_50_ for ostracods is 301–715 µg•L^−1^ (Sanchez-Bayo and Goka 2006) and cladocerans can tolerate much higher concentrations, with 48-h LC_50_s up to 45 mg•L^−1^ (Hayasaka et al. 2012). However, the threshold for sublethal effects is much lower than for lethal effects. Morrissey et al. (2015), in a review of published data on 49 species of insects and crustaceans, noted that the mobility of many aquatic invertebrates is impacted by acute exposure to imidicloprid concentrations of 1 µg•L^−1^ or lower and chronic exposure to concentrations of 0.1 µg•L^−1^. Based on the most sensitive species, they suggested that long term chronic concentrations greater than 0.035 µg•L^−1^ would have deleterious impacts on aquatic communities. Given that levels of contamination often exceed this value, concern has grown over the potential harm that imidacloprid and other nicotinoids pose to non-target organisms through sublethal effects on reproduction, behavior, physiology, and development (e.g., Drobne et al. 2008; Lukancic et al. 2010; Gonalons and Farina 2015; Chaimanee et al. 2016), and through the indirect effects of habitat alteration and predator/prey dynamics (reviewed in Gibbons et al. 2015).

In North America, crayfish are commonly occurring, non-target organisms vulnerable to imidacloprid contamination. Found in a wide range of freshwater habitats, including streams, rivers, lakes, ponds, sloughs, wet meadows, and ground water, they comprise a highly diverse assemblage, with 350 species found in the United States, mostly in the southeast (Smith 2001), compared to the currently recognized five species in Europe (Kouba et al. 2014). When present, crayfish are the largest arthropods in their communities, and they have substantial ecological impacts— facilitating leaf litter breakdown (Schofield et al. 2001), modifying benthic sediments through bioturbation (Statzner et al. 2000), and functioning as both predators and prey (Momot 1992; Usio and Townsend 2004). However, in spite of this, crayfish are remarkably underrepresented in investigations of nicotinoid pesticides, underscored by the fact that recent reviews of the impacts on non-target organisms do not mention any studies that have investigated the impacts of imidacloprid on crayfish (Wood and Goulson 2017; Morrissey et al. 2015; Pisa et al. 2015). We were able to locate only one research publication that directly investigated the impacts of a nicotinoid on a crayfish species, showing that the 48-h LC_50_ acute toxicity endpoint for *Orconectes propinquus* exposed to clothianidin was 805 µg•L^−1^ (Miles et al. 2017).

While crayfish should be investigated due to their ecological importance, they are also attractive subjects for ecotoxicology research because of their long history as model organisms in behavioral and neurological research. They have been widely used in studies of the neurochemistry of aggression and social dominance (Huber et al. 1997a, b; Tierney and Mangiamele 2001, Fong and Ford 2014) and of the neuro-muscular control of body stances and escape responses (reviewed in Edwards et al. 1999). Stereotypical behaviors, like the crayfish tail flip escape response, a reflex triggered by visual and mechanosensory inputs, are well understood (Edwards et al. 1999). Also, specific body postures have been identified and are tracked in behavioral studies, like the meral spread (raised body and outspread claws) and abdomen extensions seen during fights with conspecifics to establish social dominance (Watanabe et al. 2016). In natural populations, defensive body postures and behaviors are critically important for survival as they allow individuals to obtain mates and to defend shelters and developing embryos (Figler et al. 1995, 1997, 1999). In ecotoxicology investigations of pesticide exposed animals, defensive postures and behaviors are relevant because they are mediated by neuro-muscular systems that may be targeted by toxicants, resulting in deleterious fitness consequences.

The effect of imidacloprid on the neuromuscular systems of other arthropods raises the concern that crayfish defensive behaviors could be impacted. The carabid *Platynus assimilis*, which feeds on crop pests, was at first hyperactive and then hypoactive after feeding on sublethal doses of the nicotinoid thiamethoxam (Tooming et al. 2017). Honeybees (*Apis mellifera*) exposed to sublethal doses of imidacloprid showed impaired motor-functioning and could not maintain normal postures or readily right themselves (Williamson et al. 2014, Lunardi et al. 2017). While the mechanism is not known, it has been hypothesized that imidacloprid binds to neonicotinic acetylcholine receptors (nAChRs) in the honey bee abdominal nerve cord (Williamson et al. 2014). If this is also true of crayfish, then we might see similarly impaired motor functioning.

To determine if sublethal doses of imidacloprid impact the tail flip response and other behaviors involving motor functioning in crayfish, like claw raising and pinching, we presented a threatening stimulus to crayfish exposed to environmentally relevant concentrations of imidacloprid to determine if they had a decreased response compared to control animals. We also investigated the righting ability of imidacloprid exposed animals compared to controls.

## Materials and methods

### Specimen collection and imidacloprid treatments

We collected male and female *Orconectes rusticus* opportunistically from Stony Brook River in South Hadley, Massachusetts, USA (42.258032°N, 72.571630°W) in November of 2015, and housed them individually in plastic containers with 2 L of river water at room temperature (approximately 25 °C) in the laboratory. Crayfish were housed and tested in water from the collection site, as we have found that field-caught animals are more likely to survive in the laboratory when they are kept in water from their natural habitat. We did not measure the imidacloprid concentrations in Stony Brook at the time of this study. However, the water for the study was collected in late fall when pesticides are not used locally, and imidacloprid levels were checked in January of 2018, after the study took place, by injecting a 500 µL sample into a liquid chromatography tandem-mass spectrometer (LC-MS/MS; Agilent 6460) (see Jansson and Kreuger 2010). In January of 2018, the background imidacloprid concentration at this site was 0.015 µg•L^−1^, about half the level identified by Morrissey et al. (2015) as the threshold for sublethal effects on the most sensitive aquatic invertebrate species. Also, stock solutions using river water yielded expected concentrations when checked by LC-MS/MS—for example, an average concentration of 10.7 mg•L^−1^ was obtained for 10 mg•L^−1^ stock solution mixed from Stony Brook river water in January 2018.

Each crayfish was fed a pellet of Xenopus Nutrient (Connecticut Valley Biological) every other day. Identifying marks were painted onto the dorsal cephalothorax with nail polish, and carapace length and claw length were measured with digital calipers (±0.01 mm). Seven crayfish each were randomly assigned to river water control (trace imidacloprid), 1, 10, and 100 µg•L^−1^ imidacloprid treatments; however, one individual in the 10 µg•L^−1^ treatment lost a claw after the first day and was removed from the experiment, leaving six individuals in this treatment.

### The rod test (a threatening stimulus assay)

We used the rod test to present a threatening stimulus to crayfish: a 1 min acclimation period was followed by taps with a glass rod into the crayfish’s container at a 90 degree angle from the bottom and about 0.5 cm directly in front of the crayfish. A single assay included the acclimation period and 10 taps, each lasting 2 s, with a 10 s rest between each tap while the rod was outside of the water and the crayfish’s line of sight. Crayfish remained in their own containers for the assay. Responses were scored on a scale of one to five, from least to most aggressive (c.f., Figler et al. 1995): 1 = tail flip, 2 = back away, 3 = no response (neutral), 4 = lift and spread claws and/or move forward, 5 = pinch the rod. Responses to the 10 taps were averaged at the conclusion of each assay to obtain a single data point for each crayfish. The rod test was performed three days before imidacloprid exposure to assess the baseline condition and then repeated every other day after the first day of exposure for a total of five rod test assays during the 10 day pesticide exposure period.

### Righting ability experiment

We conducted a single trial of the righting ability experiment after 24 days of imidacloprid exposure using the same specimens and treatments from the rod test experiment. We examined the righting ability of each crayfish by placing it on its back and timing how long it took to flip itself back over. Each trial lasted 30 s. Immediate flips were scored as 1 s, and crayfish that did not right themselves by the end of the trial were given a score of 30 s. Seven crayfish were used for the control, 1, and 10 µg•L^−1^ treatments in the righting ability experiment because the crayfish with the missing claw was included. However, the 100 µg•L^−1^ treatment included just six crayfish because one individual was lost from this treatment group after the rod assay experiment was completed.

### Statistical analyses

We used a one-way ANOVA to compare crayfish carapace lengths and claw sizes across treatments to ensure that crayfish were evenly distributed by size. A *T*-test was used to compare the average responses of female and male crayfish in the baseline rod assay to ensure that crayfish sex did not influence the behavioral scores.

To determine if imidacloprid concentration and the duration of exposure were associated with a decreased behavioral response to the rod (the threatening stimulus) during the rod test assays, a two-way, repeated measures ANOVA was used. The frequency of the neutral response (no response to the rod) exhibited by crayfish during the rod tests was the response variable. Independent variables were pesticide treatment (four levels: control with trace pesticide, 1, 10, and 100 µg•L^−1^), assay number (six levels: baseline rod test, followed by rod test assays every other day for the 10 day exposure period), and the interaction between pesticide treatment and assay number. Crayfish identity was a random factor.

Once we found that pesticide treatment, assay number (duration of exposure), and the interaction of these independent variables all significantly impacted the frequency of the neutral response, we sought to determine when during the 10 day experiment differences arose in crayfish behavioral responses between pesticide treatments. To do this, we used a one-way ANOVA to compare the frequency of the neutral response between pesticide treatments on each assay day. When the ANOVA test yielded significant results, a Dunn-Bonferroni procedure was used for post-hoc pairwise comparisons.

The assumptions for all ANOVA tests investigating the neutral response were met. Neutral response residuals were assessed using P-P plots and a frequency histogram, and were found to be normally distributed. Also, the assumption of sphericity for the repeated measures ANOVA was met (Mauchley’s test of sphericity, *p* > 0.05).

Finally, we compared the righting times of crayfish across pesticide treatments with the non-parametric Kruskal Wallis H test because the data for crayfish righting times were not normally distributed and data transformations were not able to resolve this issue. Following the Kruskal Wallis H test, pairwise comparisons were made using Dunn-Bonferroni post hoc tests. IBM SPSS Statistics 22.0 was used for all statistical analyses.

## Results

### Crayfish size and sex

Average crayfish carapace length was 25.2 ± 0.9 mm ($\overline x$ ± s.e.; *n* = 27) and the average claw length was 20.7 ± 1.0 mm; there were no significant differences in either body size measure for crayfish in the pesticide treatments (claw length, *F*_3,23_ = 0.14, *p* = 0.94; carapace length, *F*_3,23_ = 0.058, *p* = 0.98). And while there were fewer females (*n* = 7) than males (*n* = 20) in the study, females were evenly distributed across treatments groups, with one or two individuals per treatment, and showed similar levels of aggression compared to males. Average baseline scores for the rod test (threatening stimulus) were 2.7 ± 0.11 ($\overline x$ ± s.e.; *n* = 20) for males and 2.7 ± 0.41 ($\overline x$ ± s.e.; *n* = 7) for females, which were not significantly different (t = 0.07, d.f. = 7, *p* = 0.95).

### The effects of pesticide concentration and duration of exposure on defensive behaviors

**Fig. 1:**
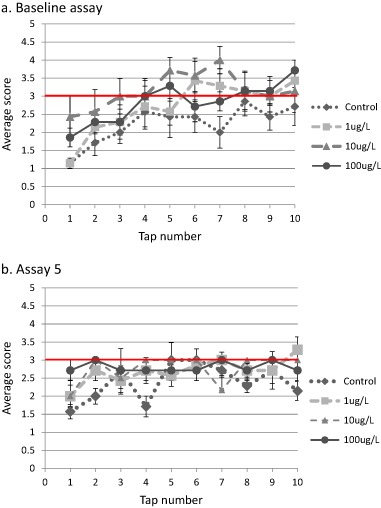
*Orconectes rusticus* behavioral responses to a threatening stimulus in the rod test. **a** Pre-exposure baseline assay (corresponds to assay 0 on the x-axis in Fig. 2). **b** final assay after 10 days of imidacloprid exposure. Scores of 1–2 indicate withdrawal, 3 is the neutral response (demarked with a red line), and 4–5 indicate aggressive responses. For each panel, data are $\overline x$ ± s.e.; *n* = 7 except for the 10 µg•L^−1^ treatment with *n* = 6

In the rod test, which measured crayfish responses to 10 successive taps, crayfish tended to back or tail-flip away from the rod during the first few taps, but then became more neutral to aggressive in their responses to the rod by the 10th tap. These responses were especially apparent during the baseline assay and appeared to show that crayfish were habituating to the rod (Fig. 1a). By the last day of the experiment, after 10 days of exposure to imidacloprid (Fig. 1b), habituation was less pronounced, especially for crayfish exposed to 100 µg•L^−1^ imidacloprid which showed a high frequency of neutral responses throughout the rod test.

**Fig. 2:**
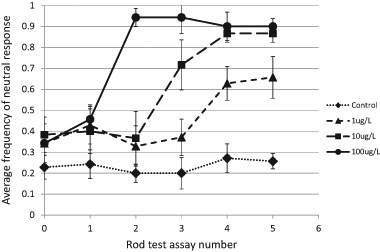
Average frequency of the neutral response (behavior score = 3) in the rod test for four concentrations of imidacloprid over 10 days. The x-axis indicates the rod test assays which were conducted one day before imidacloprid exposure to assess the baseline condition and then repeated every other day, thereafter. Assay 1 = 2 days of exposure, assay 2 = 4 days, and so on. Error bars correspond to mean ± s.e.; *n* = 7 for each treatment except for the 10 µg•L^−1^ treatment with *n* = 6

To determine if imidacloprid exposure was associated with a decreased behavioral response to a potential threat, we calculated the average frequency of the neutral response (a score of three on the behavioral response scale) out of the 10 rod taps administered to each crayfish during the rod test. We found a highly significant interaction (*F*_15,115_ = 4.279, *p* < 0.001) between pesticide concentration and duration of exposure, where crayfish exposed to higher concentrations of imidacloprid began to show a greater frequency of the neutral response after shorter exposure durations compared to those exposed to lower concentrations (Fig. 2).

During the pre-exposure, baseline rod test (assay 0, Fig. 2), the frequency of the neutral response was 0.23 ± 0.06 ($\overline x$ ± *s.e*.; *n* = 7), 0.34 ± 0.09 (*n* = 7), 0.38 ± 0.09 (*n* = 6), and 0.34 ± 0.05 (*n* = 7) for the control, 1, 10, and 100 µg•L^−1^ imidacloprid treatments, respectively. These were not significantly different response frequencies (F_3,23_ = 0.822, *p* = 0.5). After 2 days of pesticide exposure (assay 1, Fig. 2), there were still no significant differences in the rod test assays (F_3,23_ = 1.017, *p* = 0.4). However, after four days of exposure (assay 2, Fig. 2), crayfish in the 100 µg•L^−1^ treatment rarely responded to the rod, with a neutral response frequency of 0.94 ± 0.03 ($\overline x$ ± *s.e*.; *n* = 7), compared to 0.2 ± 0.04 (*n* = 7), 0.33 ± 0.10 (*n* = 6), and 0.37 ± 0.13 (*n* = 7) for the control, 1, and 10 µg•L^−1^ treatments, respectively. Thus, on day four, the 100 µg•L^−1^ treatment crayfish were significantly less responsive to a threatening stimulus compared to those in the other treatments (F_3,23_ = 17.4, *p* < 0.001; 100 neutral response frequency > control,1,10, *p* < 0.001).

After 6 days of exposure (assay 3, Fig. 2), crayfish in both of the highest pesticide treatments, 10 and 100, were significantly less responsive (i.e., had higher neutral response frequencies) than those in the control and 1 µg•L^−1^ imidacloprid treatments (F_3,23_ = 17.5, *p* < 0.001; 100,10 > control,1, *p* < 0.05). Finally, after eight days of pesticide exposure (assay 4, Fig. 2), all crayfish exposed to imidacloprid were significantly less responsive to the threatening stimulus compared to controls (F_3,23_ = 18.3, *p* < 0.001; 100,10,1 > control, *p* = 0.006). And this remained true on the last day of the experiment (assay 5, Fig. 2) (F_3,23_ = 21.9, *p* < 0.001; 100,10,1 > control, *p* = 0.001) when the neutral response frequencies were 0.26 ± 0.04 ($\overline x$ ± s.e.; *n* = 7), 0.66 ± 0.10 (*n* = 7), 0.87 ± 0.04 (*n* = 6), and 0.90 ± 0.05 (*n* = 7) for the control, 1, 10, and 100 µg•L^−1^ imidacloprid treatments, respectively.

### The impact of pesticide treatment on righting ability

**Fig. 3:**
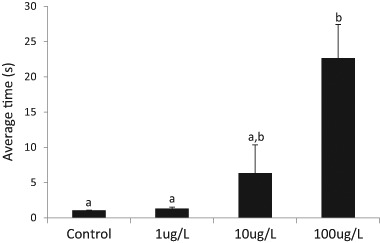
Average time (s) for crayfish to right themselves when placed on their backs after exposure to imidacloprid for 24 d. Error bars correspond to $\overline x$ ± s.e.; *n* = 7 for each treatment except for the 100 µg•L^−1^ treatment with *n* = 6. Shared letters above the bars indicate no significant difference according to the Dunn-Bonferroni post hocanalysis

When we placed crayfish from the four treatment groups on their backs after 24 days of imidacloprid exposure, we found large differences in the time they took to right themselves (H(3) = 15.524, *p* = 0.001; Fig. 3). Four out of the six crayfish in the 100 µg•L^−1^ treatment did not right themselves during the 30 sec trial period, while only one crayfish in the 10 µg•L^−1^ group did not right itself during the trial period. All of the crayfish in the 0 µg•L^−1^ and 1 µg•L^−1^ treatments righted themselves immediately or after a few seconds. The very large variation in righting times in the 100 µg•L^−1^ exposed crayfish indicated that some individuals in this group were much more impaired in their ability to right themselves than others, and it also resulted in data distribution differences between pesticide treatment groups, a violation of the Kruskal Wallis H test that could not be corrected. Pairwise comparisons (which should be interpreted cautiously) showed statistically significant differences in the righting times between the 100 µg•L^−1^ treatment group crayfish (mean rank = 23.67) and the control crayfish (mean rank = 8.86) (*p* = 0.005), and between the 100 and 1 µg•L^−1^ crayfish (mean rank = 9.43) (*p* = 0.005). There were no significant differences in righting times for the other comparisons: control vs. 1 µg•L^−1^ (*p* = 1.0), control vs. 10 (mean rank = 15.43) (*p* = 0.646), 1 vs. 10 (*p* = 0.851), and 10 vs. 100 (*p* = 0.316).

## Discussion

To determine if exposure to imidacloprid reduces defensive behaviors in the Rusty crayfish *Orconectes rusticus*, we subjected individual crayfish to the rod test (a threatening stimulus assay) several times over a 10-day experimental period while exposing them to one of three nominal imidacloprid concentrations. We assessed the defensive behaviors of the control and pesticide exposed crayfish in our study by tapping a glass rod repeatedly in front of them. In the baseline assays for all treatment groups, crayfish often tail-flipped or backed away from the first tap, but either did not respond to the rod or were slightly aggressive towards it in the last few taps. This decreased response meets a primary criterion for habituation, where the decrease in a behavioral response to a repeated stimulus resembles an exponential function (*c.f*., Thompson and Spencer 1966). Habituation in the tail flip response towards a threat has been documented in intact crayfish (Krasne and Woodsmall 1969; Wine et al. 1975) and is understood at the cellular level, where repeated electrical stimulation reduces the excitability of the lateral giant (LG) interneurons that interface with the motor neurons causing abdominal flexion (Araki and Nagayama 2005; Nagayama and Araki 2015). Our behavioral scoring system included the tail flip response, but also captured a spectrum of behaviors, ranging from escape to aggression, all of which showed habituation to repeated threats.

The majority of our crayfish showed habitation to the stimulus in the baseline rod test; however, those exposed to high pesticide concentrations or to lower concentrations for a longer duration showed a greatly reduced response to the rod, overall. Crayfish on the highest dose also had difficulty righting themselves when displaced onto their backs. Our behavioral observations are consistent with what has been found for other arthropods exposed to sublethal concentrations of nicotinoid pesticides. For example, bees fed sucrose concentrations with environmentally relevant concentrations of imidacloprid showed movement coordination problems, difficulty righting, and hypoactivity (Suchail et al. 2001; Williamson et al. 2014). And sublethal concentrations of imidacloprid have been found to negatively impact burrowing and feeding in the aquatic larvae of a midge and caddisfly species (Pestana et al. 2009), reduce leaf shredding in the amphipod, *Gammaras pulex* (Agatz et al. 2014), and reduce self-grooming behaviors in a leaf cutting ant (Galvanho et al. 2013).

After 10 days of imidacloprid exposure, we found that crayfish exposed to imidacloprid were less likely to respond to a threat compared to control crayfish with no added pesticide. Also, the damage appeared progressive: at the highest concentration, crayfish showed impaired responses after about four days, but crayfish were equally impaired after an additional two days at the second-highest dose. Chronic toxicity for nicotinoids has been described in bees, where imidacloprid concentrations 60–6000 times lower than the acute dose were found to have the same toxic effects within a few days (Suchail et al. 2001). For mayfly nymphs, the LC_50_ chronic dose is 40–100 times lower than the LC_50_ acute dose (van den Brink 2016); and low chronic doses of nicotinoids were found to be equivalent to higher acute doses in several other arthropod species (Stoughton et al. 2008; Roessink et al. 2013). While the mechanism for chronic toxicity has not been established, it is known that imidacloprid binds to nicotinic acetylcholine receptors (nAChRs), possibly irreversibly (Tennekes and Sanchez-Bayo 2013), where it acts as an agonist (Brown et al. 2006) and potentially causes neuron damage and death from excitotoxicity (Rondeau et al. 2014). Wild crayfish are potentially vulnerable to acute exposure immediately following crop applications of nicotinoid pesticides in habitats in or near agricultural areas; however, chronic toxicity may be a more common problem since, with the exception of rice cultivation, nicotinoid pesticides are not generally introduced directly to aquatic habitats.

In the field, rusty crayfish could be impacted by sublethal concentrations of nicotinoids in several ways. Crayfish rely on claw raising threats, tail-flipping, and fighting to defend themselves from predators and to acquire and maintain control of shelters (Davis and Huber 2007). We found that crayfish exposed to the lowest concentrations of nicotinoid pesticide were impaired in these behaviors, while those exposed to higher concentrations did not respond to threats at all, showed a stiff body posture, and often could not right themselves when they were displaced onto their backs. Stiff, unresponsive bodies pose a particular challenge in streams and rivers: Beketov and Liess (2008) showed that sub-lethal concentrations of nicotinoids caused downstream drift in several species of benthic macroinvertebrates, presumably because they could no longer grip the substratum. Rusty crayfish, which are territorial and return to previously occupied shelters after feeding (Davis and Huber 2007), could be particularly vulnerable to displacement by downstream drift which would move them away from their shelters into unknown and potentially suboptimal habitats.

Only a few other studies have investigated the sublethal effects of nicotinoid pesticides on crayfish. The northern clear water crayfish *Orconectes propinquus* was found to be less responsive to a tap on the carapace when exposed to clothianidin concentrations greater than 0.05 ppm (Miles et al. 2017). Barbee and Stout (2009) found that the red crayfish *Procambarus clarkii* became hyper-aggressive when exposed to sublethal concentrations of thiamethoxam above 500 µg•L^−1^ but they saw no changes in the behavior of crayfish exposed to sublethal doses of thiamethoxam (<500 µg•L^−^1), clothianidin, and dinotifuran (no amount given). However, because these were anecdotal observations and they did not perform any behavioral assays, they may not have noticed other changes in behavior resulting from sublethal doses that would impact survival in the wild. These results together with our findings suggest that crayfish species, type of nicotinoid, and pesticide concentration all influence the severity of impact. Crayfish are among the largest macroinvertebrates in freshwater ecosystems, where they play important ecological roles, and they are also food for humans; as such, their unintended exposure to nicotinoid pesticides is concerning.
